# Molecular study of Nucleophosmin 1(NPM1) gene in acute myeloid leukemia in Kurdish population

**DOI:** 10.4314/ahs.v21i2.26

**Published:** 2021-06

**Authors:** Galawezh Obaid Othman, Nawsherwan Sadiq Mohammad, Chiman Hameed Saeed

**Affiliations:** 1 Salahuddin University, Education College. Biology Dep; 2 Hawler Medical University, College of Medicine. Nanakaly Teaching Hospital for Blood Diseases. Erbil- Iraq; 3 Hawler Medical University. Medical Research Center. Erbil-Iraq

**Keywords:** Acute myeloid leukemia, Nucleophosmin-1 (NPM-1) gene mutation, PCR

## Abstract

**Background:**

In patients with Acute Myeloid Leukemia (AML) the most frequent acquired molecular abnormalities and important prognostic indicators is nucleophosmin-1 (NPM1) mutations. Our study aims was molecular study of Nucleophosmin -1 gene in Acute Myeloid Leukemia in Kurdish population.

**Patients &Methods:**

A total of 50 patients with AML, (36) of them attended Nanakaly Hospital and (14) attended Hiwa Hospital and 30 healthy subjects as control were selected randomly, all were matched of age and gender. Polymerase chain reaction (PCR) was used for detection of NPM1 gene mutation. Three samples of PCR product for NPM1 gene mutations were sequenced, and mutations were determined by comparison with the normal NPM1 sequence NCBI (GenBank accession number NM_002520).

**Results:**

Out of 50 patients with AML, 5 (10%) of them were NPM1 gene mutation positive, and 45 (90%) were negative. The mutation were a base substitution (C to A), (G to C), (G to T), transversion mutation in addition of frame shift mutation and all mutated cases were heterozygous and retained a wild type allele.

**Conclusion:**

Identification of NPM1 mutations in AML are important for prognostication, treatment decision and optimization of patient care.

## Introduction

Malignant transformation of immature hematopoietic cells through a complex multistep process that requires cooperation of different types of genetic alterations lead to develop Acute Myeloid Leukemia (AML) and itis the most common acute leukemia in adults which increases with age. Classification of AML subtype is contribute to the development of new therapeutic approaches.^1^ The most frequently mutated genes in AML is Nucleophosmin-1 (NPM1) which found in nearly one-third of newly diagnosed cases both in younger and older adults.^2^ Nucleophosmin-1 (NPM1) defined as a nucleus cytoplasm shuttling protein that is ubiquitously expressed and is highly conserved and this protein has been shown to contribute to many basic cellular processes such as ribosomes biosynthesis, regulation of centrosome function, genome stability, DNA duplication, transcriptional regulation. and preventing aggregation of proteins in the nucleolus, in addition it participates in (ARF- P53) tumor suppressor pathway.^1^ Recent study found that in human there is a gene, located on chromosome 5q35, contains 12 exons ranging in size from 58 to 358 bp called NPM1 gene which is a multifunctional phosphoprotein, localized primarily to the granular regions of the nucleolus.^3^ In acute myeloid leukemia (AML) genetic markers are important for knowledge on pathophysiology, choice of specific treatment and risk classification.^4^ In NPM1 gene frameshift mutation at exon 12 that interferes with cell cycle regulation is an alternative leukemogenetic mechanism rather than chromosomal translocations occur frequently in AML patients an insertion into exon 12 of NPM1 leads to an aberrant localization of the protein in the cytoplasm^1^,^5^, which blocks the differentiation of myeloid cells through gain of function for the AML phenotype.^6^ Moreover, tumorigenic effect can confer due to impairment of NPM1 function and this because of deletion or dislocation or the enhancement of NPM1 function due to overexpression.^5^

Understanding the molecular mechanisms by which mutant NPM1 promotes leukemogenesis and for the maintenance of leukemia, identification of NPM1 gene mutations might help clinician to provide proper treatment for the patients.^2^ This study is designed to evaluate Nucleophosmin-1(NPM1) gene in Acute Myeloid Leukemia in Kurdish population.

## Patients & methods

### Blood Sampling

The studied patients included 50 patients with AML 20 (40%) were male and 30 (60%) were female) of whom (36) attended Nanakaly Hospital and (14) patients attended Hiwa hospital together with 30 apparently healthy subjects as control selected randomly who were of comparable age and sex. Forty-one patients were diagnosed as de novo AML and 2 patients were diagnosed as secondary AML evolving from preexisting MDS. Forty two patients were newly diagnosed, and 8 patients were in relapse. Twenty were male and (30) were female their ages ranged between (1–97 years). Clinical data including present, past, and family history were registered. The study has been approved by ethical committee of the College of Education, Salahuddin University. From each patient and control, 2 ml of EDTA anticoagulated venous blood was obtained for DNA Extraction in Hawler Medical University/Research Center Laboratory. The Tubes for DNA study were kept in deep freeze (-20oC) until the day of analysis. Peripheral blood, bone marrow aspirate and biopsy smears of the patients were examined in addition to immunophenotypic study by at least two hematology consultants for diagnosis of AML and their sub classification according to FAB classification.

### DNA Extraction and Detection of NPMI Mutation

All samples were analyzed for NPMI mutation in exon 12 using PCR method. Mononuclear cells were isolated from the leukemia samples by centrifugation and genomic DNA extracted according to the kit protocol (Bioneer).

### Sample amplification

The polymerase chain reaction (PCR) amplification of the NPM1 gene exon 12 fragments was performed with the following oligonucleotide primers: forward (NPM11-F) (5′-ACCACATTTCTTTTTTTTTTTTTCCAGGCT-3′) and reverse (NPM12-R) (5′-CCTGGACAACATTTAT CAAACACGGTA-3′) One and half µl of primers and 1.5 µl of genomic DNA was amplified in a 50 µl reaction mixture contain (47 µl) of one PCRTM which is a ready to use PCR reaction mixture (is a pre mixed solution containing Taq DNA polymerase, PCR buffer, dNTPs, gel loading dyes and novel green, It contains the fluorescence dye which is directly detected on the blue light transilluminator or UV epi-illuminator after the DNA electrophoreses. A positive reaction was assessed in duplicate and a negative control was included in each reaction. PCR amplification was performed using PCR Thermal cycler ,Amplification process consisted of 2 min at 95°C for denaturation 35 cycles of 1 min at 94°C for denaturation, 30 sec at 61°C for annealing, 1 minute at 72°C for extension and 7 minutes at 72°C for the final extension.

### GEL Electrophoresis

Ten µl of the PCR product was electrophoresed on 2.5% agarose gel (Bioneer), using 100 bp DNA ladder. The one MARK 100 with novel green was optimized for direct loading on to unstained agarose gels. The ladders provide highest level of convenience during the routine handling and avoid commonly used gel staining procedures with ethidium bromide.

### DNA Sequencing and Nucleotide Alignment

Three samples of PCR product for NPMI gene mutations was sequenced, the sampls was sent to the Genomic institute by lab in Hawler.

## Results

**Figure 1:**
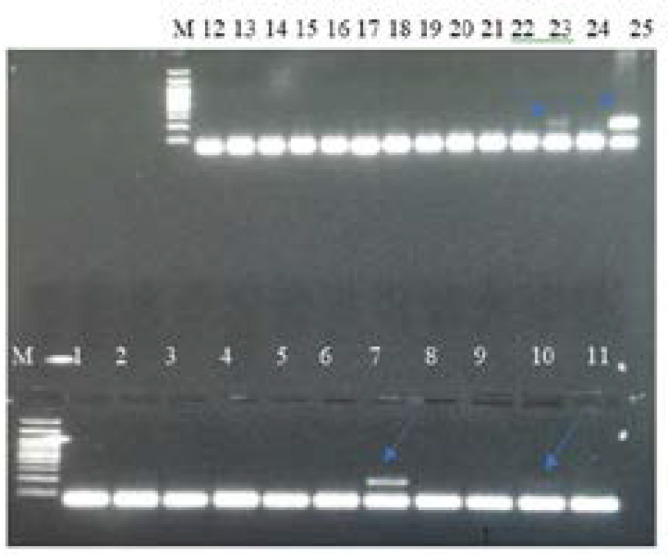
PCR product on 2.5% agarose gel electrophoresis for detection of *NPMI* mutation in adult AML patients. Lane 1 and 2: Amplified product from healthy control. Lane 7,10,23 and 25 amplified products from patients show extra mutated band of *NPMI.* Other Lanes amplified product from patients' wild type (about 133 bp). M: marker.

This study showed male to female ratio 1:1.5. The mean age of cases was (36.47) years, peak age incidence was in the sixth decade forming 10 (20%) cases followed by first and third decades forming 8 (16 %) cases of each. Out of 50 patients with AML 5 (10%) of them were NPMI mutation positive, and 45 (90%) were negative. (Figure 1).

### DNA Sequencing and Nucleotide Alignment

NPM1mutation was detected in 5 out of 50 cases (10%). Three samples were randomly selected from 5 positive samples for sequence analysis of NPM1mutation. the mutations were determined by comparison with the normal NPM1 sequence (GenBank accession number NM_002520) and a normal control that was included in each run sequence alignment was performed with Clustal W (using default settings) in MEGA 4.0.2 software). Different sequence variants were observed, all leading to a frame shift in the region encoding the C-terminal of the NPM1 protein. All mutations consisted of insertion. For our results to be certified also by manual using the normal sequence of NPM1 with Exons. Indeed, the results confirmed the identity of the NPMI gene isolated from the patient's samples with that in NCBI as shown in figure (2).

**Figure:**
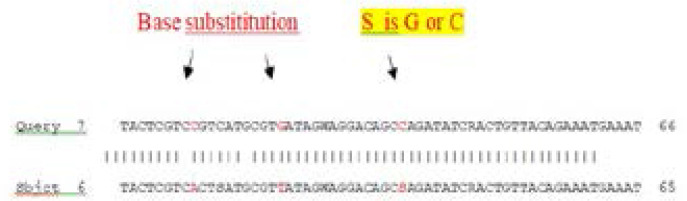


The results show that there was a base substitution (C to A), (G to C) and (G to T) transversion mutation. While S is G or C according to nucleotide ambiguity code, and Y is C to T NM_002520.6 Homo sapiens nucleophosmin 1 (NPM1), transcript variant mRNA. In our study all mutated cases were heterozygous and retained a wild type allele. All mutations consisted of insertion, deletion and substitution.

**Figure:**
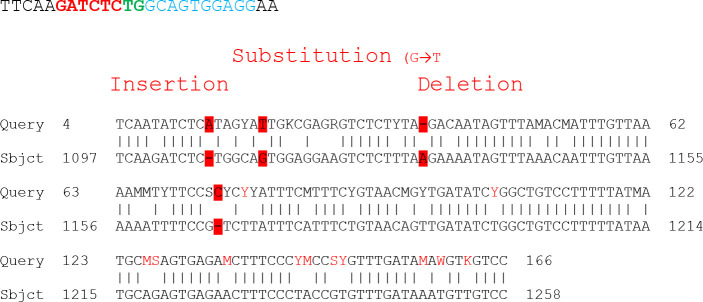


The results show that there were many types of mutation in DNA sequence represent insertion of nitrogen base A just before the coding region which is represent splice- site mutation and affected the transcription factor of this exon and failure of transcription and translation, also a base substitution (G to T), transversion mutation, deletion of nitrogen base A which leading to truncated proteins. Also, another type of mutation was observed in this sequence represent insertion of nitrogen base C in coding region which leading to frameshift mutation and finally changes all codons.

While the letters M represent nitrogen base C or A, S represent G or C, K represent T or G, W represent A or T and finally Y represent C or T

**Figure:**
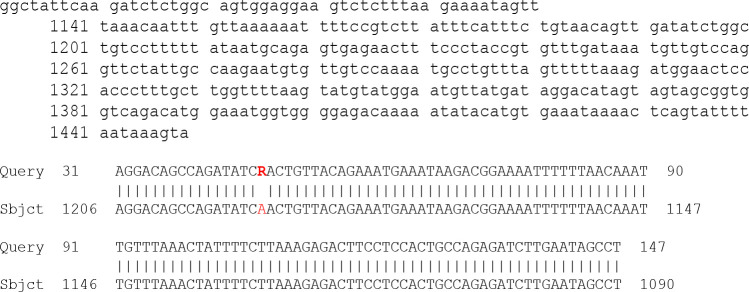


The results of the above sequence shown that R represent A or G

**Figure:**
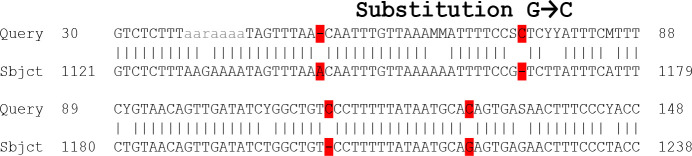


The results shown that base substitution G to C leading to change the amino acid and A, C insertion mutation in the same sequence which is leading to frameshift mutation.

**Figure:**
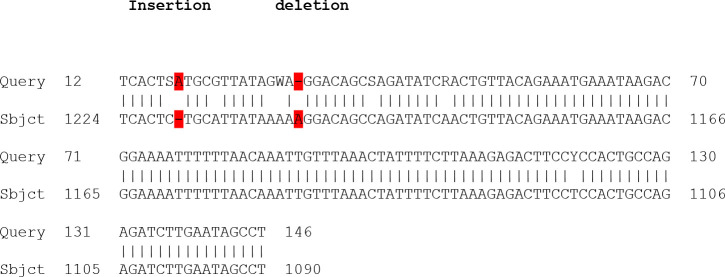


## Discussion

Acute myeloid leukemia (AML) is a heterogeneous group of clonal disorders and it is believed that this heterogeneity is related to molecular abnormalities that alter normal cellular mechanisms of self-renewal, proliferation, and differentiation, notably, it is fatal disease with dismal outcomes. Even after achieving initial remission, most patients relapse and ultimately succumb to their disease.^7^

Abelson and his Colleagues in their study evaluated the incidence of acute myeloid leukaemia (AML) and they found that it increases with age and 90% of mortality exceeds when diagnosed after age 65.^8^ Kouchkovsky and Abdul-Hay in his study estimated the incidence of AML and found that the incidence increases with age, from 1.3 per 100 000 population in patients less than 65 years old, to 12.2 cases per 100 000 population in those over 65 years and he observed that 70% of patients 65 years or older will die of their disease within 1 year of diagnosis.^9^ Previous study when analyzed 44 patients' samples with normal karyotype-AML investigated that the median age of the patients was 62 years (range 18–89 years).^10^

The age and cytogenetics have been most important prognostic criteria in AML. Recently, molecular genetics were added as another important criteria, this means identify potential targets for treatment^7^.

In present study our patients with AML 10% of them were had NPMI mutation positive, and 90% were negative. Yusoff et al. said that the most common genetic abnormality in AML were NPM1 mutations at exon 12 and he found it in approximately 24 to 45% of all AML cases.^11^ Gallagher in his study found that more than 50% of normal karyotype-AML cases harbor mutations in the NPM1 gene, and he concluded that it the highest incidence of any mutation in AML.^12^ Mencia-Trinchant, et al. found that half of his patients with AML have normal cytogenetics whereas 40% to 50% of these patients have NPM1 mutations.^7^ Balatzenko et al. detected that 24.8% of AML patients harboring NPM1-A mutation.^13^ Kunchala et al. reported that patients with normal karyotype-AML carry NPM1 mutations estimated approximately 50–60% that characterized by cytoplasmic dislocation of the NPM1 protein.^6^ A good molecular marker for assessing the clinical status and predicting the outcomes in AML patients was Nucleophosmin 1 gene (NPM1) mutation.^14^

Webersinke et al. reported that as primary leukemogenic the mutations in exon 12 of the nucleophosmin (NPM1) gene have been described and event in up to 35% of adult acute myelogenous leukemia (AML) cases and they added that this type of AML is listed as provisional entity in the World Health Organization classification of tumors of the hematopoietic and lymphoid tissues.^15^ Juliusson and Colleagues reported that mutations in Nucleophosmin 1 (NPM1) provide prognostic information with clinical relevance through choice of treatment and they found that significantly females more often had NPM1 mutation than male.^4^ Dohner et al. found that AML patients with concurrent NPM1 mutation were older and more frequently female.^16^

In present study the nucleotide sequence was read by Bio Edit Program and use of Blast in NCBI to compare with other isoforms of NPMI in Gene Bank. Our results represented that there was a base substitution (C to A), (G to C) and (G to T), transversion mutation, deletion of nitrogen base A which leading to truncated proteins, in addition the results showed that there were many types of mutation in DNA sequence represent insertion of nitrogen base A just before the coding region which is represent splice- site mutation that affected the transcription factor of this exon and failure of transcription and translation. Also, another type of mutation was observed in this sequence represent insertion of nitrogen base C in coding region which leading to frame shift mutation and finally change all codons this means that in our study all mutated cases were heterozygous and retained a wild type allele.

Brunetti et al. found that more than 50 mutations in exon 12 in NPM1 which consist of 4 bp insertions and the most common is mutation A which found in about 80% of cases, they clarified that all exn 12 variants cause a frameshift in the last few C-terminal amino acids of NPM1.2 Mencia-Trinchant et al. they also indicated that all possible four nucleotide insertions in exon 12 frameshift insertion mutations and 100% of DNA fragments contain NPM1 insertions which is type A.7 Jeon et al. in his study identified NPM1 mutations in 19 (22.9%) of the 83 AML patients and 16 (84.2%) of them were type A NPM1 mutations.^17^

The “type A” mutation is defined by insertion of the four nucleotides thymine, cytosine, thymine and guanine and results in a lengthening of the protein. Pastore et al. found in their study that the majority of cases frameshift NPM1 mutations due to an insertion of four bases, which cluster in exon 12 and according to the inserted tetranucleotide they mention that in 80% the most common being type A mutations (TCTG), followed by type B (CATG) and type D (CCTG) mutations in about 10%.^18^

B23 protein also known as Nucleophosmin 1 (NPM1), resides primarily in the nucleus, but shuttles continuously between the nucleus and cytoplasm and this protein contains a number of motifs that predominantly oligomeric and binds to other proteins, including tumor suppressor proteins. Notably, mutations in NPM1 can be detected in AML at relapse, even many years after the initial diagnosis, so that these mutations recently updated World Health Organization (WHO) classification of myeloid neoplasms and acute leukemia and has been defined as a distinct molecular leukemia in addition it is a reliable biomarker for assessment of disease status in AML.^3^

## Conclusion

We concluded that molecular study of Nucleophosmin 1 (NPM1) gene mutation important because the abnormalities in NPM1 play a critical role in several types of myeloid malignancies and investigation of NPM1 gene mutation in AML patients can be used as diagnostic tools for prognostication and optimization of patient care as well as open the door to new therapeutic strategies for cancer therapy.
